# Evaluation of the Widal tube agglutination test for the diagnosis of typhoid fever among children admitted to a rural hdospital in Tanzania and a comparison with previous studies

**DOI:** 10.1186/1471-2334-10-180

**Published:** 2010-06-22

**Authors:** Benedikt Ley, George Mtove, Kamala Thriemer, Ben Amos, Lorenz von Seidlein, Ilse Hendriksen, Abraham Mwambuli, Aikande Shoo, Rajabu Malahiyo, Shaali M Ame, Deok R Kim, Leon R Ochiai, John D Clemens, Hugh Reyburn, Harald Wilfing, Stephen Magesa, Jacqueline L Deen

**Affiliations:** 1 International Vaccine Institute, Seoul, Korea; 2 National Institute for Medical Research - Amani Centre, Tanga, Tanzania; 3 Joint Malaria Program, Tanga, Tanzania; 4 Teule Hospital, Muheza, Tanga, Tanzania; 5 Mahidol Oxford Research Unit, Bangkok, Thailand; 6 Public Health Laboratory (Pemba) - Ivo de Carneri, Chake Chake, Tanzania; 7 London School of Hygiene and Tropical Medicine, Keppel St, London, UK; 8 University of Vienna, Biocenter, Vienna, Austria

## Abstract

**Background:**

The diagnosis of typhoid fever is confirmed by culture of *Salmonella enterica *serotype Typhi (*S. typhi*). However, a more rapid, simpler, and cheaper diagnostic method would be very useful especially in developing countries. The Widal test is widely used in Africa but little information exists about its reliability.

**Methods:**

We assessed the performance of the Widal tube agglutination test among febrile hospitalized Tanzanian children. We calculated the sensitivity, specificity, positive predictive value (PPV), and negative predictive value (NPV) of various anti-TH and -TO titers using culture-confirmed typhoid fever cases as the "true positives" and all other febrile children with blood culture negative for *S. typhi *as the "true negatives."

**Results:**

We found that 16 (1%) of 1,680 children had culture-proven typhoid fever. A single anti-TH titer of 1:80 and higher was the optimal indicator of typhoid fever. This had a sensitivity of 75%, specificity of 98%, NPV of 100%, but PPV was only 26%. We compared our main findings with those from previous studies.

**Conclusion:**

Among febrile hospitalized Tanzanian children with a low prevalence of typhoid fever, a Widal titer of ≥ 1:80 performed well in terms of sensitivity, specificity, and NPV. However a test with improved PPV that is similarly easy to apply and cost-efficient is desirable.

## Background

*Salmonella enterica *serotype Typhi (*S. typhi*), the causative agent of typhoid fever, was calculated to have caused approximately 200,000 deaths globally in 2000 [1]. The clinical picture of typhoid fever is nonspecific; confirmed diagnosis through blood or bone-marrow culture requires expensive and labor-intensive isolation and identification of the organism, which may take up to seven days. A cheap and rapid alternative laboratory test is desirable, especially for developing country settings where typhoid fever is a major public health burden.

Various agglutination tests have been developed [2] of which the Widal method is the oldest and remains the most widely used. The test was first introduced by F. Widal in 1896 [2] and is based on a macroscopically visible serum - mediated agglutination reaction between *S. typhi *somatic lipopolysacharide O antigens (TO) and flagellar H antigens (TH). Laboratories in industrialized countries have stopped performing the assay. In Africa the Widal test is still widely used [3] because typhoid fever is perceived to be endemic in the area [3] and the Widal test is the only rapid diagnostic assay that is available and affordable. The Widal test is commonly performed when children and adults present with fever to treatment centers, as few centers have the capacity to perform micro-bacterial culture [4]. Despite this widespread use, little has been published on its performance in Africa.

We assessed the sensitivity, specificity, positive predictive value (PPV), and negative predictive value (NPV) of the Widal tube agglutination test among Tanzanian children hospitalized with febrile illness and compared our results with those from previous studies.

## Methods

### Study site and population

The study was conducted as part of a childhood fever surveillance study at Teule Hospital in Muheza district of Northeastern Tanzania from 2008 to 2009. Muheza district is located between the foothills of Kilimanjaro and the coastal town of Tanga. The area is highly endemic for *Plasmodium falciparum *malaria with perennial transmission and two seasonal peaks [5]. HIV sero-prevalence among antenatal clinic attendees was about 7% in 2007 [6]. Teule Hospital is a busy 330-bed district-level general hospital, serving a surrounding population of 277,000. It has two 35-bed in-patient pediatric wards receiving approximately 5,000 child admissions per year (2008).

### Inclusion Criteria

Children aged 2 months to 14 years were screened for eligibility during study hours from 7am to 7pm, Monday to Sunday. Children with fever of 3 or more days prior to admission, or fever of less than 3 days but with at least one severity criteria (respiratory distress, deep breathing, respiratory distress in combination with severe pallor, prostration, capillary refill ≥ 3 seconds, temperature gradient, systolic blood pressure < 70 mm Hg, coma defined by Glasgow Coma Scale ≤ 10 or Blantyre Coma Scale ≤ 2, severe jaundice, history of 2 or more convulsions in the last 24 hours, blood glucose < 3 mmol associated with clinical signs, neck stiffness, bulging fontanel, or oxygen saturation < 90%) were recruited into the study. All clinical information was recorded on a standard case record form. Treatment was provided according to national guidelines. On admission we collected 3 to 5 milliliters (ml) of blood (depending on body weight) from each eligible child for the Widal test and a single blood culture. All clinical procedures were performed by trained study clinical officers and nurses under the supervision of study physicians.

### Laboratory

Blood for culture was inoculated into BacT/ALERT™ Pediatric-fan bottles (bioMérieux, Marcy l'Etoile, France). Inoculated blood culture bottles were transported immediately to the hospital laboratory and incubated in the BacT/ALERT 3 D automated microbial detection system. Blood cultures were processed according to standard methods. Colonies with biochemical reactions on API20E suggestive of *Salmonellae *were confirmed serologically by slide and tube agglutination testing using specific O and H antisera (Becton Dickinson, NJ, USA).

A minimum of 0.5 ml of blood was separated to obtain serum samples. All serum samples were frozen at -70°C until Widal testing was done in three batches. Widal testing was performed using standardized TO (IgM and IgG) and TH (IgG) antigens (Stained *Salmonella *Antigens kit, Span Diagnostics, India) according to standard methods as described on the package insert. In brief, each sample was diluted to a concentration of 1:40 with 0.9% NaCl in two separate plastic tubes. A single drop of antigen was added to the respective tube. Incubation times for both O and H agglutinations were 16 to 20 hours at 37°C in a water bath. Evaluation of test results was performed by at least two lab technicians on an independent basis under standardized light conditions. If agglutination was detected in a sample, testing was done on that sample diluted serially from 1:80 to 1:1280 for both O and H antigens. All laboratory procedures were performed by trained laboratory technicians under the supervision of microbiologists. Technicians performing the Widal tests were blinded to the participants' clinical picture and blood culture results.

### Data management

Case report forms were double-entered into custom-made data entry programs using MS-Access (Microsoft Corp.). Data management programs included error, range, and consistency check programs. Analyses were performed using EpiInfo v 3.4.3 (Centers for Disease Control and Prevention, USA) and Stata TM v 10.0 (Stata Corp., USA).

### Definitions and analysis

Fever was defined as stated history or presence of fever of ≥37.5°C. Bacteremia was defined as fever with isolation of pathogenic bacteria from blood culture. Children with a febrile illness were classified as follows: those with *S. typhi *subsequently isolated from blood culture (group 1), those with non-Typhi serotypes of *S. enterica *(NTS) subsequently isolated from blood culture (group 2), those with pathogenic bacteria other than *Salmonellae *subsequently isolated from blood culture (group 3), and those whose blood culture yielded no bacterial pathogen (group 4). Malaria status was not considered in the classification. In areas of high transmission of *P. falciparum *where individuals develop immunity from previous episodes of malaria starting at a young age, asymptomatic parasitemia is common and may be detected during febrile episodes caused by another infection [7,8].

For the primary analysis, sensitivity (true-positive rate) was defined as the probability that the Widal test result would be positive when blood culture confirmed that typhoid fever was present (group 1) and specificity (true-negative rate) was the probability that the Widal test result would be negative when *S. typhi *was not isolated from blood culture (groups 2, 3, and 4). The positive predictive value was the probability that culture-confirmed typhoid was present when the test was positive, and the negative predictive value was the probability that culture-confirmed typhoid was not present when the test was negative. Since serological tests detect antibody response and perform better after a period of time from the onset of the illness, sensitivity, specificity, PPV, and NPV were also calculated separately for cases presenting with fever for 5 days or less and for more than 5 days. Because controversy exists about what is the most appropriate control group to use [9-11], we conducted a secondary analysis using two alternative "true-negative" control groups as follows: those with NTS and other pathogenic bacteria isolated from blood culture (groups 2 and 3), and those with pathogenic bacteria other than *Salmonellae *isolated from blood culture (group 3).

Comparisons were made using the Chi square or Fishers' Exact test, as appropriate. Sensitivity, specificity, PPV, and NPV were calculated according to standard methods. The 95% confidence interval for sensitivity and specificity was calculated using the Wilson's Score method [12]. Analyses were performed using EpiInfo v 3.4.3 (Centers for Disease Control and Prevention, USA) and Stata TM v 10.0 (Stata Corp., USA).

### **Literature review**

We conducted a literature review to compare our main findings with those from previous studies of similar character. We included studies of the Widal test which were identified by direct searches of the MEDLINE database through PubMed. The searches were restricted to publications from 1993 to date. We also conducted supplementary searches of the references in retrieved articles. Abstracts were reviewed and if relevant, the article was included.

### Ethics

The fever surveillance was conducted following the principles governing biomedical research involving human subjects. Prior written informed consent was obtained from the parent or guardian of all study participants. The study protocol was approved by the National Institute of Medical Research, Tanzania, and the International Vaccine Institute Institutional Review Board.

## Results

**Figure 1:**
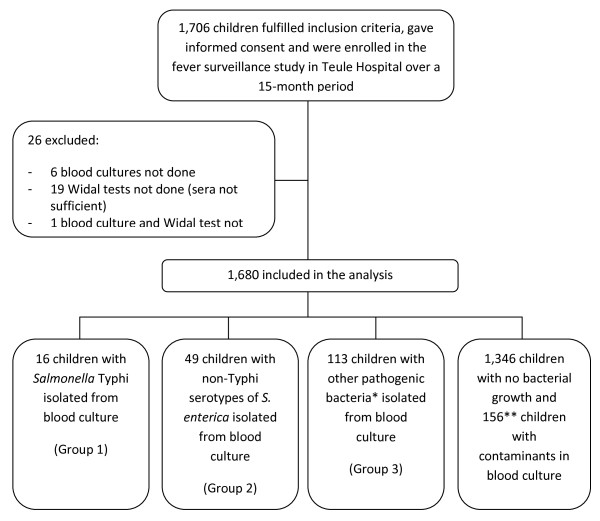
**Flow of patients**. *Species included: *Streptococcus pneumonia *(n = 11), beta hemolytic Streptococci (n = 10), *Staphylococcus aureus *(n = 5), *Haemophilus influenzae *type b (n = 26), *Escherichia coli *(n = 31), *Acinetobacter species *(n = 6), Non-fermenters (n = 12), Others: (n = 5), *Haemophilus parainfluenzae *(n = 2), and Gram negative rods not identified (n = 5). **Species included: Bacillus (n = 22), Diphtheroids (n = 7), Micrococcus (n = 9), alpha-hemolytic Streptococcus viridans (n = 4), coagulase negative Staphylococcus (n = 104), yeast (n = 5), mixed bacterial species (n = 4), Gram positive rods not identified (n = 1).

The flow of patients is shown in Figure 1. A total of 1,706 febrile children were enrolled out of which 26 (1.5%) were excluded for the following reasons: 19 or 1.1% had no blood culture done, 6 or 0.4% had no Widal testing done due to insufficient quantities of sera, and one case or 0.1% had no blood culture nor Widal testing done. A total of 1,680 (98.5%) samples were included in the analysis. There were 16 or 1.0% culture-confirmed typhoid fever cases (group 1), 49 or 2.9% with NTS infection (group 2), and 113 or 6.7% with non-*Salmonella *bacteremia (group 3). From 1,502 (89.4%) children, no pathogenic bacteria were isolated from blood culture (group 4).

We assessed the age distribution and highest anti-TH and -TO titer by blood culture-confirmed diagnosis (Table 1). Children with typhoid fever were significantly older compared to the other groups. Anti-TH agglutination titers of 1:80 and higher were detected among 12/16 (75.0%) culture-confirmed typhoid fever cases compared to 7/49 (14.3%) of those with NTS infection, and 1/113 (0.9%) with other bacteremia (p values = 0.001 and < 0.001, respectively). Similarly, anti-TO agglutination titers of 1:80 and higher were detected among 11/16 (68.8%) cases of typhoid fever compared to 7/49 (14.3%) of those with NTS infection and 0/113 (0%) with other bacteremia (p values < 0.001 and < 0.001, respectively).

**Table 1:** Number and cumulative frequencies of *a*nti-TH and anti-TO levels overall and by blood culture isolates

Highest titer reached; Number (%)	All (n = 1,680)	Children with culture-confirmed typhoid fever (n = 16)	Children with non-Typhi serotypes of *S. enterica *(n = 49)	Children with other pathogenic bacteria (n = 113)	Children with no pathogenic bacteria isolated (n = 1,502)
Median age (Range)*	1.83 (14.81)	7.21 (11.96)	1.58 (6.73)	1.43 (11.83)	1.84 (14.81)

**Anti-TH**					

≥1:640	15 (0.9)	3 (18.8)	5 (10.2)	0 (0)	7 (0.5)
1:320	24 (1.4)	6 (37.5)	6 (12.2)	0 (0)	12 (0.8)
1:160	36 (2.1)	11 (68.8)	7 (14.3)	0 (0)	18 (1.2)
1:80	46 (2.7)	12 (75.0)	7 (14.3)	1 (0.9)	26 (1.7)
1:40	85 (5.1)	12 (75.0)	9 (18.4)	3 (2.7)	61 (4.1)
No agglutination	1,595 (94.9)	4 (25.0)	40 (81.6)	110 (97.3)	1,441 (95.9)

**Anti-TO**					

≥1:640	6 (0.4)	3 (18.8)	2 (4.1)	0 (0)	1 (0.1)
1:320	18 (1.1)	6 (37.5)	3 (6.1)	0 (0)	9 (0.6)
1:160	34 (2.0)	10 (62.5)	6 (12.2)	0 (0)	18 (1.2)
1:80	44 (2.6)	11 (68.8)	7 (14.3)	0 (0)	26 (1.7)
1:40	95 (5.7)	12 (75.0)	10 (20.4)	3 (2.7)	70 (4.7)
No agglutination	1,585 (94.3)	4 (25.0)	39 (79.6)	110 (97.3)	1,432 (95.3)

*P values: Group 1 vs 4 < 0.001; Group 2 vs 4 = 0.209; Group 3 vs 4 = 0.013

### Primary analysis

We calculated the sensitivity, specificity, PPV, and NPV of various Widal test cut-offs for the diagnosis of typhoid fever (Table 2). The sensitivity, specificity, PPV, and NPV of an anti-TH titer of 1:80 were 75, 98, 26, and 100%, respectively. The sensitivity, specificity, PPV, and NPV of an anti-TO titer of 1:80 were 69, 98, 25, and 100%, respectively.

**Table 2:** Primary analysis for the performance* of the Widal test for typhoid fever diagnosis (n = 1680)

Widal titer	Sensitivity (95%CI)	Specificity (95%CI)	Positive Predictive Value	Negative Predictive Value
TH ≥1:80	12/16, 0.75 (0.51-0.90)	1630/1664, 0.98 (0.97-0.99)	12/46, 0.26	1630/1634, 1.00
TH ≥1:160	11/16, 0.69 (0.44-0.86)	1639/1664, 0.98 (0.98-0.99)	11/36, 0.31	1639/1644, 1.00
TH ≥1:320	6/16, 0.38 (0.18-0.61)	1646/1664, 0.99 (0.98-0.99)	6/24, 0.25	1646/1656, 0.99
TO ≥1:80	11/16, 0.69 (0.44-0.86)	1631/1664, 0.98 (0.97-0.99)	11/44, 0.25	1631/1636, 1.00
TO ≥1:160	10/16, 0.63 (0.39-0.82)	1640/1664, 0.99 (0.98-0.99)	10/34, 0.29	1640/1646, 1.00
TO ≥1:320	6/16, 0.38 (0.18-0.61)	1652/1664, 0.99 (0.99-1.00)	6/18, 0.33	1652/1662, 0.99

*The values were calculated using culture-confirmed typhoid fever cases (group 1; n = 16) as the true positives and those cases from which *S*. typhi were not isolated from blood culture (groups 2, 3, and 4; n = 1664) as the true negatives.

We compared the performance of the Widal test between patients who presented with fever of 5 days or less and those who presented with more than 5 days of fever (Table 3). Of the 16 typhoid fever cases, 6 (37.5%) presented with fever of 5 days or less, and 10 (62.5%) with more than 5 days of fever. Of the 1,664 children in the control group, 1,117 (67%) presented with fever of 5 days or less and 544 (33%) with more than 5 days of fever. Three control cases, whose fever duration was unknown, were excluded from the analysis. The sensitivity of an anti-TH and -TO titer of 1:80 increased, however not significantly, from 67% to 80% and 67% to 70%, respectively, with the longer duration of fever prior to admission (both p > 0.05). The PPV of an anti-TH and -TO titer of 1:80 increased from 21% to 30% and 19% to 30% (both p > 0.05), respectively, with the longer duration of fever prior to admission. But the change was also not statistically significant.

**Table 3:** Comparison of the performance* of the Widal test for typhoid fever diagnosis by number of days of fever** prior to admission

Widal titer	Sensitivity (95%CI)	Specificity (95%CI)	Positive Predictive Value	Negative Predictive Value
**≤ 5 days fever (n = 1123)**				

TH ≥1:80	4/6, 0.67 (0.30-0.90)	1102/1117, 0.99 (0.98-0.99)	4/19, 0.21	1102/1104, 1.00
TH ≥1:160	3/6, 0.50 (0.19-0.81)	1106/1117, 0.99 (0.98-0.99)	3/14, 0.21	1106/1109, 1.00
TH ≥1:320	2/6, 0.33 (0.10-0.70)	1110/1117, 0.99 (0.99-1.00)	2/9, 0.22	1110/1114, 1.00
TO ≥1:80	4/6, 0.67 (0.30-0.90)	1100/1117, 0.98 (0.98-0.99)	4/21, 0.19	1100/1102, 1.00
TO ≥1:160	3/6, 0.50 (0.19-0.81)	1107/1117, 0.99 (0.98-1.00)	3/13, 0.23	1107/1110, 1.00
TO ≥1:320	1/6, 0.17 (0.03-0.56)	1112/1117, 1.00 (0.99-1.00)	1/6, 0.17	1112/1117, 1.00

**> 5 days fever (n = 554)**				

TH ≥1:80	8/10, 0.80 (0.49-0.94)	525/544, 0.97 (0.95-0.98)	8/27, 0.30	525/527, 1.00
TH ≥1:160	8/10, 0.80 (0.49-0.94)	530/544, 0.97 (0.96-0.98)	8/22, 0.36	530/532, 1.00
TH ≥1:320	4/10, 0.40 (0.17-0.69)	533/544, 0.98 (0.96-0.99)	4/15, 0.27	533/539, 0.99
TO ≥1:80	7/10, 0.70 (0.40-0.89)	528/544, 0.97 (0.95-0.98)	7/23, 0.30	528/531, 0.99
TO ≥1:160	7/10, 0.70 (0.40-0.89)	530/544, 0.97 (0.96-0.98)	7/21, 0.33	530/533, 0.99
TO ≥1:320	5/10, 0.50 (0.24-0.76)	537/544, 0.99 (0.97-0.99)	5/12, 0.42	537/542, 0.99

*The values were calculated using culture-confirmed typhoid fever cases (group 1; n = 16) as the true positives and those cases from which *S*. typhi were not isolated from blood culture (groups 2, 3, and 4; n = 1664) as the true negatives. **Three patients whose fever duration was unknown were excluded from the analysis.

### Secondary analysis

Using different control groups, we compared the resulting sensitivity, specificity, PPV, and NPV of a Widal test cut-off of an anti-TH and -TO titer of ≥1:80 for the diagnosis of typhoid fever (Table 4). Changing the control group had no significant effect on the sensitivity, specificity, and NPV but markedly increased the PPV of an anti-TH titer of ≥1:80 from 26% to 92% and the PPV of an anti-TO titer of ≥1:80 from 25% to 100%.

**Table 4:** Secondary analysis of the performance of a Widal anti-TH and -TO titer of ≥1:80 for typhoid fever diagnosis using group 1 as true positives and three different control groups as true negatives*

Control group	Sensitivity (95%CI)	Specificity (95%CI)	Positive Predictive Value	Negative Predictive Value
**TH titer ≥1:80:**				

Groups 2, 3, and 4 (n = 1664)	12/16, 0.75 (0.51-0.90)	1630/1664, 0.98 (0.97-0.99)	12/46, 0.26	1630/1634, 1.00
Groups 2 and 3 (n = 162)	12/16, 0.75 (0.51-0.90)	154/162, 0.95 (0.91-0.97)	12/20, 0.60	154/158, 0.97
Group 3 (n = 113)	12/16, 0.75 (0.51-0.90)	112/113, 0.99 (0.95-1.00)	12/13, 0.92	112/116, 0.97

**TO titer ≥1:80:**				

Groups 2, 3, and 4 (n = 1664)	11/16, 0.69 (0.44-0.86)	1631/1664, 0.98 (0.97-0.99)	11/44, 0.25	1631/1636, 1.00
Groups 2 and 3 (n = 162)	11/16, 0.69 (0.44-0.86)	155/162, 0.96 (0.91-0.98)	11/18, 0.61	155/160, 0.97
Group 3 (n = 113)	11/16, 0.69 (0.44-0.86)	113/113, 1.00 (0.97-1.00)	11/11, 1.00	113/118, 0.96

*Group 1 were those with *S*. typhi isolated from blood culture (n = 16), group 2 were those with non-typhi serotypes of *S. enterica *(NTS) isolated from blood culture (n = 49), group 3 were those with pathogenic bacteria other than *Salmonellae *isolated from blood culture (n = 113), and group 4 were those whose blood culture yielded no bacterial pathogen (n = 1502).

### Comparison with earlier studies

We found 4 articles from 3 countries. In this series, the age group included and prevalence of blood-culture confirmed typhoid fever varied considerably. The cut-off titer used ranged from ≥1:20 to ≥1:200 and the resulting sensitivity, specificity, PPV and NPV varied considerably (Table 5).

**Table 5:** Summary of Widal performances in earlier studies

Authors	Date	Study Country	Sample Size	Age classes included	Prevalence of S. typhi in participants	Sensitivity	Specificity	PPV	NPV	Cut Off Titer	Control Group(s)	Gold Standard
**Choo et al**.	1993	Malaysia	2382	Children	6.1%	89%	89%	< 50%	99.2%	O or H ≥1:40	Non-typhoid febrile children admitted to hospital	Blood Culture

**Parry et al**.	1999	Vietnam	2000	Children & Adults	30.8%	O: 49% H: 67%; O or H ≥1:100:88%	O: 97% H: 96%; O or H ≥1:100:87%	O: 88% H: 88%; O or H ≥1:100:74%	O: 82% H: 87%; O or H ≥1:100:94%	O:≥1:200 H: ≥1:100; O or H ≥1:100	Lab confirmed malaria, dengue or bacteremia	Blood Culture

**Wilke et al**.	2002	Turkey	410	≥18 y	13.2%	52% Post 7-10 d: 90%	88% Post 7-10 d: 90%	76% Post 7-10 d: 88%	71% Post 7-10 d: 93%	O: ≥1:200 H: ≥1:200	Healthy controls, nontyphoidal febrile patients, blood culture negative febrile cases	Blood Culture, Stool Culture

**Olsen et al**.	2004	Vietnam	80	≥3y	73.8%	64% (field) 61% (lab)	76% (field) 100% (lab)	88% (field) 100% (lab)	43% (field) 48% (lab)	O or H ≥1:100	Lab confirmed bacteremia, AFB, dengue, malaria, pos. stool culture, pos. urine culture	Blood Culture

**Ley et al**.	This study	Tanzania	1680	2 m. - 14y	1%	75%	98%	26%	100%	H: ≥1:80	Non-typhoid febrile children admitted to hospital	Blood Culture

## Discussion

We found that a Widal titer of ≥1:80 was the optimal indicator of typhoid fever in our study population. The PPV, NPV and specificity in the primary analysis was more-or-less unchanged from the cut-off titers of ≥1:80 to ≥1:320, whereas the sensitivity was highest at a cut-off titer of ≥1:80. Although the Widal test at this cut-off titer performed relatively well in terms of sensitivity, specificity and NPV, its PPV was low. It has been argued that PPV is the most important measure of a clinical diagnostic method since it represents the proportion of patients with positive test results that are correctly diagnosed [13]. The PPV is not intrinsic to the test; it is affected by prevalence of the disease. In our setting, where 16 (1%) out of 1,680 febrile patients admitted to the pediatric ward had culture-proven typhoid fever, a negative Widal test result would have a good predictive value for the absence of disease but a positive result would have a low predictive value for typhoid fever, making the use of the Widal test in our setting questionable.

In a previous paper describing the clinical aspects of the children included in this study [14] older age and long duration of fever were predictive of typhoid fever in this group.

There are several difficulties associated with evaluation of the Widal test. Firstly, levels of agglutinins detectable in the non-infected populations of different areas vary considerably by time and place depending on the endemicity of the disease, which affects test performance. For example, the sensitivity and specificity of a Widal test anti-TO titer of 1:80 in Kolkata, India was 58% and 85% [10] compared to our findings of 69% and 98%. Secondly, test performance is also affected by cross-reacting infections. In our study, none of the 113 children with non-*Salmonella *bacteremia exhibited titers above 1:80 for both O and H, although cross-reactions with *Klebsiella spp*. and *Staphylococcus aureus *[15] have been reported. In contrast, 7 (14.3%) of the 49 children with NTS had titers above 1:80 for both O and H. There is also the possibility of cross-reactivity with non-bacterial infections such as malaria, dengue, hepatitis A, and infectious mononucleosis [2,9,16]. The third limitation is the choice of a satisfactory gold standard for diagnosis. We used blood culture-positive patients as our true positives. Although bone marrow culture would be the ideal gold standard, this test is difficult to perform in small rural hospitals in Africa. We found that 26 (1.7%) of 1,502 children from whom pathogenic bacteria were not isolated showed agglutination at 1:80 or higher, both for O and H antigens. These may be Widal false positive results due to cross-reaction. Alternatively, since the reported sensitivity of a single blood culture is only 40% to 60% [16-19], some of these are likely to be false negative blood culture results. The final, and what we found to be the most contentious issue, is the selection of the most appropriate control group. It is difficult to choose patients with febrile illness who are blood culture-negative and who definitely do not have typhoid fever. Furthermore, there were relatively few hospitalized children with no bacteremia in the same age range as those with typhoid fever. Thus, the control children were significantly younger than the cases. For our primary analysis, we used groups 2, 3 and 4 (i.e., all children admitted for a febrile illness who were subsequently culture-negative for *S. typhi*). These would be the most conservative controls for specificity since blood culture picks up only a fraction of typhoid cases, resulting in a control group that is likely contaminated with culture-negative typhoid cases. Despite this, the specificity of the Widal test was high. Using the more exclusive control groups as others had done previously [9-11] did not appreciably alter the sensitivity, specificity, and NPV but they increased the PPV.

The previous studies included in our review (Table 5) had not been performed in Africa hence different cut - off titers were applied, and the resulting sensitivity, specificity, PPV and NPV varied considerably. PPV as well as NPV are dependent on the prevalence of disease within the group of participants; the selection process of study participants has therefore direct influence on the results. The difficulty of choosing the correct control group has been noted earlier [9]. While the gold standard, blood culture, is applied in most studies, the true negatives may be defined as febrile patients with a non-typhi laboratory-confirmed diagnosis as done by Parry et al. and Olsen et al. [9,20]. Alternatively, some studies use healthy controls. Choo et al [21]. considered all febrile cases with an *S.typhi *negative blood culture as the control group which is problematic as a number of blood culture-negative results are likely to be false-negative due to the poor sensitivity of the blood culture [17-19,22]. Furthermore, it is difficult to compare the different test kits, as varying antigens perform differently [23].

## Conclusion

In summary, a Widal titer of ≥ 1:80 performed relatively well in terms of sensitivity and specificity. However, the low prevalence of typhoid fever of approximately 1% amongst children at Teule Hospital meant that the Widal test was only useful for excluding the disease.

Considering the low cost of Widal testing and the absence of comparably cheap tests, Widal testing is likely to remain the test of choice in many developing country settings. But the need for rapid and cheap diagnostic tools with superior performance remains high.

## Competing interests

The authors declare that they have no competing interests.

## Authors' contributions

BL performed the Widal tests, analyzed and compared results and wrote the manuscript; GM was in charge of the implementation and management of the study; KT performed Widal tests, analyzed results and contributed to the manuscript; BA supervised the laboratory where blood cultures were performed and contributed to the manuscript; LvS provided scientific support to study staff and manuscript and was involved in clinical care of participants; IH was involved in clinical care of participants; AM was in charge of data management; AS performed blood culture procedures, RM facilitated activities to make data collection possible, SA provided laboratory support; DRK performed the statistical analysis; RLO provided scientific support to the manuscript; JDC provided scientific support to the manuscript; HR provided major contributions to the manuscript; HW provided scientific support to the manuscript; SM facilitated activities to make data collection possible; JLD provided major scientific support to the manuscript and was involved in clinical care of participants.

All authors have read and approved the final manuscript

## Pre-publication history

The pre-publication history for this paper can be accessed here:

http://www.biomedcentral.com/1471-2334/10/180/prepub
